# Supplementary material for: Analysis of symbiotic backscatter empowered wireless sensors network with short-packet communications
Source: PLoS One. 2024 Aug 26;19(8):e0307366. doi: 10.1371/journal.pone.0307366 (PMC11346738; doi:10.1371/journal.pone.0307366)
Supplement: Attachment — Submitted filename: Response to Reviewers.pdf [file pone.0307366.s002.pdf]

Response to Reviewers

Original Manuscript ID: **PONE-D-24-11836**
Original Article Title: **Analysis of Symbiotic Backscatter Empowered Wireless Sensors Network with Short-Packet Communications**
To: The Editor of PLOS ONE
Re: Response to reviewers

Dear Editors,

Thank you for allowing us to revise our manuscript. We have carefully considered all the Reviewers' comments and made the following key updates:

- Figure 1 has been renewed to prevent copyright issues.
- Two references have been added in accordance with the Reviewers' suggestions.
- Appropriate explanations and clarifications have been included to address the Reviewers' concerns about Eq. (16).

We are uploading a revised manuscript with changes highlighted in blue, along with point-by-point responses to the comments.
Furthermore, we request that our article be assigned to the same set of reviewers for further review. We look forward to receiving your feedback.

Sincerely,
Quang Vinh Do, Bui Vu Minh, Quang-Sang Nguyen, Byung-seo Kim

Journal requirements

Point P 0.1 — *Please ensure that your manuscript meets PLOS ONE's style requirements, including those for file naming. The PLOS ONE style templates can be found at https://journals.plos.org/plosone/s/file?id=wjVg/PLOSOne_formatting_sample_main_body.pdf and https://journals.plos.org/plosone/s/file?id=ba62/PLOSOne˙formatting_sample_title_authors_affiliations.pdf*

Response: We confirm this requirement.

Point P 0.2 — *Please note that PLOS ONE has specific guidelines on code sharing for submissions in which author-generated code underpins the findings in the manuscript. In these cases, all author-generated code must be made available without restrictions upon publication of the work. Please review our guidelines at https://journals.plos.org/plosone/s/materials-and-software-sharing#loc-sharing-code and ensure that your code is shared in a way that follows best practice and facilitates reproducibility and reuse.*

Response: We confirm this requirement.

Point P 0.3 — *Thank you for stating the following financial disclosure:*

"This work was supported by the National Research Foundation of Korea (NRF) grant funded by the Korea government (MSIT) (No. 2022R1A2C1003549)."

At this time, please address the following queries:

a) *Please clarify the sources of funding (financial or material support) for your study. List the grants or organizations that supported your study, including funding received from your institution.*

b) *State what role the funders took in the study. If the funders had no role in your study, please state: "The funders had no role in study design, data collection and analysis, decision to publish, or preparation of the manuscript."*

c) *If any authors received a salary from any of your funders, please state which authors and which funders.*

d) *If you did not receive any funding for this study, please state: "The authors received no specific funding for this work."*

Please include your amended statements within your cover letter; we will change the online submission form on your behalf.

Response: We clarify that the funding statement is as "*This work was supported by the National Research Foundation of Korea (NRF) grant funded by the Korea government (MSIT) (No. 2022R1A2C1003549).*"

Point P 0.4 — *We note that your Data Availability Statement is currently as follows: [All relevant data are within the manuscript and its Supporting Information files.]*

Please confirm at this time whether or not your submission contains all raw data required to replicate the results of your study. Authors must share the "minimal data set" for their submission. PLOS defines the minimal data set to consist of the data required to replicate all study findings reported in the article, as well as related metadata and methods (https://journals.plos.org/plosone/s/data-availability#loc-minimal-data-set-definition).

For example, authors should submit the following data:

- *The values behind the means, standard deviations and other measures reported;*
- *The values used to build graphs;*
- *The points extracted from images for analysis.*

Authors do not need to submit their entire data set if only a portion of the data was used in the reported study.

If your submission does not contain these data, please either upload them as Supporting Information files or deposit them to a stable, public repository and provide us with the relevant URLs, DOIs, or accession numbers. For a list of recommended repositories, please see https://journals.plos.org/plosone/s/recommended-repositories.

If there are ethical or legal restrictions on sharing a de-identified data set, please explain them in detail (e.g., data contain potentially sensitive information, data are owned by a third-party organization, etc.) and who has imposed them (e.g., an ethics committee). Please also provide contact information for a data access committee, ethics committee, or other institutional body to which data requests may be sent. If data are owned by a third party, please indicate how others may request data access.

Response: We confirm that "All relevant data are within the manuscript and its Supporting Information files." We have also provided our simulation code as *Supporting Information Files*.

Point P 0.5 — *We note that Figure 1 in your submission contain copyrighted images. All PLOS content is published under the Creative Commons Attribution License (CC BY 4.0), which means that the manuscript, images, and Supporting Information files will be freely available online, and any third party is permitted to access, download, copy, distribute, and use these materials in any way, even commercially, with proper attribution. For more*

information, see our copyright guidelines: http://journals.plos.org/plosone/s/licenses-and-copyright

We require you to either (1) present written permission from the copyright holder to publish these figures specifically under the CC BY 4.0 license, or (2) remove the figures from your submission:

1. *You may seek permission from the original copyright holder of Figure 1 to publish the content specifically under the CC BY 4.0 license. We recommend that you contact the original copyright holder with the Content Permission Form (http://journals.plos.org/plosone/s/file?id=7c09/content-permission-form.pdf) and the following text: "I request permission for the open-access journal PLOS ONE to publish XXX under the Creative Commons Attribution License (CCAL) CC BY 4.0 (http://creativecommons.org/licenses/by/4.0/). Please be aware that this license allows unrestricted use and distribution, even commercially, by third parties. Please reply and provide explicit written permission to publish XXX under a CC BY license and complete the attached form." Please upload the completed Content Permission Form or other proof of granted permissions as an "Other" file with your submission. In the figure caption of the copyrighted figure, please include the following text: "Reprinted from [ref] under a CC BY license, with permission from [name of publisher], original copyright [original copyright year]."*

2. *If you are unable to obtain permission from the original copyright holder to publish these figures under the CC BY 4.0 license or if the copyright holder's requirements are incompatible with the CC BY 4.0 license, please either i) remove the figure or ii) supply a replacement figure that complies with the CC BY 4.0 license. Please check copyright information on all replacement figures and update the figure caption with source information. If applicable, please specify in the figure caption text when a figure is similar but not identical to the original image and is therefore for illustrative purposes only.*

Response: We have replaced the figure with another.

Comments to the Author

Point P 1.1 — Is the manuscript technically sound, and do the data support the conclusions? The manuscript must describe a technically sound piece of scientific research with data that supports the conclusions. Experiments must have been conducted rigorously, with appropriate controls, replication, and sample sizes. The conclusions must be drawn appropriately based on the data presented.

Reviewer #1: Yes.

Response: Thank you.

Point P 1.2 — Has the statistical analysis been performed appropriately and rigorously?

Reviewer #1: Yes.

Response: Thank you.

Point P 1.3 — Have the authors made all data underlying the findings in their manuscript fully available?
The PLOS Data policy requires authors to make all data underlying the findings described in their manuscript fully available without restriction, with rare exception (please refer to the Data Availability Statement in the manuscript PDF file). The data should be provided as part of the manuscript or its supporting information, or deposited to a public repository. For example, in addition to summary statistics, the data points behind means, medians and variance measures should be available. If there are restrictions on publicly sharing data—e.g. participant privacy or use of data from a third party—those must be specified.

Reviewer #1: Yes.

Response: Thank you.

Point P 1.4 — Is the manuscript presented in an intelligible fashion and written in standard English?
PLOS ONE does not copyedit accepted manuscripts, so the language in submitted articles must be clear, correct, and unambiguous. Any typographical or grammatical errors should be corrected at revision, so please note any specific errors here.

Reviewer #1: Yes.

Response: Thank you.

Point P 1.5 — Review Comments to the Author

Please use the space provided to explain your answers to the questions above. You may also include additional comments for the author, including concerns about dual publication, research ethics, or publication ethics. (Please upload your review as an attachment if it exceeds 20,000 characters)

Reviewer #1: Authors investigated the feasibility of Backcom and SPC for symbiotic wireless sensor networks by analyzing the system performance. Specifically, we provide a highly approximated mathematical framework for evaluating the block-error rate (BLER) performance, followed by some useful asymptotic results.
Paper is well written and idea is clear, however I have some major comments.

1. Eq. 16 need proof.
2. Simulation and channel modelling need more detail.
3. Some important references are missing.
   a. Li et al., "Physical Layer Security for Wireless-Powered Ambient Backscatter Cooperative Communication Networks," in IEEE Transactions on Cognitive Communications and Networking, vol. 9, no. 4, pp. 927-939, Aug. 2023.
   b. `https://www.techscience.com/CMES/v138n1/54237`

Response: We would like to thank Reviewer 1 for the comments.
First, it is unfortunately that Reviewer 1 cannot capture our derivation on Eq. (16). We should reemphasize that this equation has been detail provided step by step to obtain the final equation. However, to make it more clearly, we would like to recall it as follows.

1. The average BLER at Rx has been finally provided in Eq. (11) as follows:

$$\epsilon_{\text{Rx}} \simeq \int_{0}^{\infty} \Xi\left(r_{\text{Rx}}, L_{\text{Rx}}, \gamma_{\text{Rx}}\right) f_{\gamma_{\text{Rx}}}(x)dx = \zeta_{\text{Rx}} \int_{\upsilon_{\text{Rx}}}^{\vartheta_{\text{Rx}}} F_{\gamma_{\text{Rx}}}(x)dx. \tag{11}$$

   From this equation, it is clear that if we want to obtain the BLER solution, it is required to get the CDF of γ_{Rx}, i.e., $F_{\gamma_{\text{Rx}}}(x)$.

2. To obtain the asymptotic BLER at high signal-to-noise ratio (SNR) (i.e., $\Psi \to \infty$), the simplest way here is to find the asymptotic the CDF of γ_{Rx}. Herein, we would like to recall that the asymptotic of $F_{\gamma_{\text{Rx}}}(x)$ has been derived in Eq. (15) as follows:

$$F_{\gamma_{\text{PR}}}(x) \overset{\Psi\to\infty}{\simeq} \frac{\lambda_{\text{PR}}}{\Psi}x. \tag{15}$$

3. From the above discussion, it is clear that by injecting $F_{\gamma_{\text{PR}}}(x)$ in Eq. (15) into the BLER formulation in Eq. (11), we will have the result in Eq. (16) as follows:

$$\tilde{\epsilon}_{\text{PR}} \overset{\Psi\to\infty}{\simeq} \zeta_{\text{PR}} \int\limits_{\upsilon_{\text{PR}}}^{\vartheta_{\text{PR}}} F_{\gamma_{\text{PR}}}(x)dx = \zeta_{\text{PR}} \int\limits_{\upsilon_{\text{PR}}}^{\vartheta_{\text{PR}}} \frac{\lambda_{\text{PR}}}{\Psi} x dx = \zeta_{\text{PR}} \frac{\lambda_{\text{PR}}}{2\Psi} \left[\vartheta_{\text{PR}}^2 - \upsilon_{\text{PR}}^2\right] = \lambda_{\text{PR}} \frac{2^{r_{\text{PR}}} - 1}{\Psi}. \tag{16}$$

Herein, the last step is obtained based on a basic equality that $A^2 - B^2 = (A+B)(A-B)$. Accordingly, we have that $\vartheta_{\text{PR}}^2 - \upsilon_{\text{PR}}^2 = (\vartheta_{\text{PR}} - \upsilon_{\text{PR}})(\vartheta_{\text{PR}} + \upsilon_{\text{PR}})$. Recall that $\upsilon_{\text{Rx}} = \kappa_{\text{Rx}} - 1/(2\zeta_{\text{Rx}})$ and $\vartheta_{\text{Rx}} = \kappa_{\text{Rx}} + 1/(2\zeta_{\text{Rx}})$, with $\zeta_{\text{Rx}} = \sqrt{L_{\text{Rx}}/[2\pi(2^{2r_{\text{Rx}}} - 1)]}$ and $\kappa_{\text{Rx}} = 2^{r_{\text{Rx}}} - 1$ (as provided in Eq. (10)). Following that, we can readily derive $\vartheta_{\text{PR}} + \upsilon_{\text{PR}} = 2\kappa_{\text{PR}} = 2(2^{r_{\text{PR}}} - 1)$. Similarly, we also obtain $\vartheta_{\text{PR}} - \upsilon_{\text{PR}} = 1/\zeta_{\text{PR}}$. Pulling all together, we have $\vartheta_{\text{PR}}^2 - \upsilon_{\text{PR}}^2 = 2(2^{r_{\text{PR}}} - 1)/\zeta_{\text{PR}}$. By comparing this result with the formulation of the third step derivation in Eq. (16), we can readily obtain

$$\tilde{\epsilon}_{\text{PR}} \overset{\Psi\to\infty}{\simeq} \zeta_{\text{PR}} \frac{\lambda_{\text{PR}}}{2\Psi} 2(2^{r_{\text{PR}}} - 1) = \lambda_{\text{PR}} \frac{2^{r_{\text{PR}}} - 1}{\Psi}.$$

In order to make the information to be more clearly, we have included the explanation for Eq. (16) as follows:

Sec. III, Equation 16:

Herein, the last step is obtained based on a basic equality that $x^2 - y^2 = (x+y)(x-y)$. Accordingly, we have that $\vartheta_{\text{PR}}^2 - \upsilon_{\text{PR}}^2 = (\vartheta_{\text{PR}} - \upsilon_{\text{PR}})(\vartheta_{\text{PR}} + \upsilon_{\text{PR}})$. Recall that $\upsilon_{\text{Rx}} = \kappa_{\text{Rx}} - 1/(2\zeta_{\text{Rx}})$ and $\vartheta_{\text{Rx}} = \kappa_{\text{Rx}} + 1/(2\zeta_{\text{Rx}})$, with $\zeta_{\text{Rx}} = \sqrt{L_{\text{Rx}}/[2\pi(2^{2r_{\text{Rx}}} - 1)]}$ and $\kappa_{\text{Rx}} = 2^{r_{\text{Rx}}} - 1$, as provided in Eq. (11). On that basis, we can obtain $\vartheta_{\text{PR}} + \upsilon_{\text{PR}} = 2\kappa_{\text{PR}} = 2(2^{r_{\text{PR}}} - 1)$ and $\vartheta_{\text{PR}} - \upsilon_{\text{PR}} = 1/\zeta_{\text{PR}}$. Pulling all together yields $\vartheta_{\text{PR}}^2 - \upsilon_{\text{PR}}^2 = 2(2^{r_{\text{PR}}} - 1)/\zeta_{\text{PR}}$. By comparing this result with step $(*)$ in Eq. (16), we can readily obtain the desired result.

Second, it is unfortunately that Reviewer 1 cannot find out clarification on the channel model as well as the simulation information. In this regard, we would like to recall that the description of channel model has been early provided in Section II (System Model). Specifically, it has been described before Equation (1). Meanwhile, the simulation information has been also early mentioned in Sections IV (Numerical Results and Discussions) that we use Monte-Carlo simulation method to obtain the results. Details are as follows:

- "*Under Rayleigh fading assumptions, channel gains* $|h|^2$, $h \in \{h_{PT,PR}, h_{PT,BD}, h_{PT,IR}, h_{BD,IR}\}$ *will obey exponential distributions with the parameter* $\lambda \in \{\lambda_{PT,PR}, \lambda_{PT,BD}, \lambda_{PT,IR}, \lambda_{BD,IR}\}$, *and the respective probability density function (PDF) and the cumulative distribution function (CDF) are given by [44] ..*"

- "*This section provides some illustrative numerical results using Monte-Carlo simulations to validate our developed mathematical framework. Without loss of generality, we consider* $\lambda_{PT,PR} = 2$, $\lambda_{PT,BD} = 4$, $\lambda_{PT,IR} = 3$, *and* $\lambda_{BD,IR} = 5$."

To help reader find information conveniently, we have included some information as
<u>**Sec. IV, initial paragraph:**</u>

> This section provides some illustrative numerical results using Monte-Carlo simulations to validate our developed mathematical framework, where the number of used channel realization samples is 10^4. Without loss of generality, we consider the specific parameters for Rayleigh channels as follows: $\lambda_{\text{PT,PR}} = 2$, $\lambda_{\text{PT,BD}} = 4$, $\lambda_{\text{PT,IR}} = 3$, and $\lambda_{\text{BD,IR}} = 5$ (channel modeling has been early described at Eq. (1)). Meanwhile, the key parameters related to packet designs as well as transmit SNR are details clarified per figure.

Third, we would like to emphasize that the main objective of this work is to investigate the performance of **Symbiotic Backscatter Empowered Wireless Sensors Network with Short-Packet Communications**. Herein, the focus is on Short-Packet Communication (known for Ultra-reliability and Low-latency communication applications) and Symbiotic Backscatter System. Therefore, we only select appropriate references to support the article's contribution, not including irrelevant documents. Therefore, the paper cannot cover all the literature. By checking carefully the two suggested references, both comply with the Symbiotic Backscatter System but are a bit far from the scope of this work. Specifically, these works investigated security issues for long-term communication systems. However, to improve the quality of the literature, we have included them in an appropriate introduction as follows:
<u>**Sec. I, reference [35] + [36]:**</u>

> Meanwhile, an innovative solutions for enhancing the security of low-power IoT devices using ambient backscatter communication was introduced in [35], with a strong focus on the balance between energy efficiency and security. In a very recent time, the work in [35] presented advances in enhancing the robustness of wireless communication systems against jamming attacks by designing a novel beamforming technique that utilizes the concept of symbiotic radio to effectively use the null space of interference, thereby enhancing safeguarding data transmission significantly.

We hope that Review 1 finds our clarifications and improvements satisfactory. Thank you.
